# Presenilin 1 Regulates [Ca^2+^]i and Mitochondria/ER Interaction in Cultured Rat Hippocampal Neurons

**DOI:** 10.1155/2019/7284967

**Published:** 2019-07-28

**Authors:** Eduard Korkotian, Anna Meshcheriakova, Menahem Segal

**Affiliations:** ^1^Department of Neurobiology, The Weizmann Institute, Rehovot 76100, Israel; ^2^Department of Biomolecular Sciences, The Weizmann Institute, Rehovot 76100, Israel

## Abstract

Mutations in the presenilin 1 (PS1) gene are a major trigger of familial Alzheimer's disease (AD), yet the mechanisms affected by mutated PS1 causing cognitive decline are not yet elucidated. In the present study, we compared rat hippocampal neurons in culture, transfected with PS1 or with mutant (M146V) PS1 (mPS1) plasmids in several neuronal functions. Initially, we confirmed earlier observations that mPS1-expressing neurons are endowed with fewer mature “mushroom” spines and more filopodial immature protrusions. The correlation between calcium changes in the cytosol, mitochondria, and endoplasmic reticulum (ER) is mitigated in the mPS1 neurons, tested by the response to an abrupt increase in ambient [Ca^2+^]o; cytosolic [Ca^2+^]i is higher in the mPS1 neurons but mitochondrial [Ca^2+^] is lower than in control neurons. Strikingly, mPS1-transfected neurons express higher excitability and eventual lower survival rate when exposed to the oxidative stressor, paraquat. These results highlight an impaired calcium regulation in mPS1 neurons, resulting in a reduced ability to handle oxidative stress, which may lead to cell death and AD.

## 1. Introduction

Presenilins (PSs) have raised great interest in recent years, since mutations in the genes that encode PSs are among the leading causes of familial Alzheimer's disease (fAD). Consequently, a large body of studies has attempted to decipher the role of PS in the presymptomatic phases of fAD [1–3]. One of the main leads to the involvement of PS in fAD is the association of PSs with intracellular calcium stores of the endoplasmic reticulum (ER) and the mitochondria. It has been suggested that PSs along with other proteins physically link ER with the mitochondria, at specific sites called the mitochondria-associated ER membrane (MAM, [4, 5]), where amyloid beta proteins are produced [6, 7]. There is evidence to suggest that PS1 is associated with the MAM, such that a mutant PS1 affects mitochondrial functions, including handling of excess calcium ions in the cytosol [8–12]. This provides a direct link to the proposed role of calcium handling machinery in triggering fAD, regardless of the direct molecular pathway associated with this link (e.g., the catalytic core of the gamma secretase [8, 9]). This link has been proposed already over a decade ago [12–14]. However, while it has been demonstrated that PS1 is linked to calcium handling machinery, there is still a paucity of information on the involvement of PS1 in neuronal physiology and plasticity.

A powerful tool for the analysis of the role of PS in neuronal functions is a mutant PS1 (M146V, mPS1, [12, 14]). In response to caffeine or ionomycin, activators of calcium release from the ER, mPS1 causes a larger than normal increase in free cytoplasmic [Ca^2+^]i [11, 14, 15]. Furthermore, spontaneous fluctuations of [Ca^2+^]i which are sensitive to blockers of ER calcium release are larger in mPS1 neurons than in controls [16, 17]. However, Wu et al. [18] found that conditional knockout of PS1 in mice causes a *reduction* in response of cultured hippocampal neurons to caffeine, leaving this issue still unsettled. In a different series of investigation, cultured hippocampal neurons from mPS1-knock-in mice express fewer mature dendritic spines (=mushroom spines) compared to control neurons [17] indicating that some synaptic functions are impaired in the former neurons. However, it is not clear how the observed morphological differences relate to mPS1 action.

In the present study, we explored functional differences between wild-type PS1 and mPS1-transfected cultured hippocampal neurons, using mitochondria and ER calcium sensors. Initially, we were able to replicate earlier results, but in further experiments, we suggest that aberrations in calcium exchange between mitochondria and cytosolic [Ca^2+^]i may underlie the involvement of mPS1 in the regulation of cytosolic calcium and consequently in triggering fAD.

## 2. Materials and Methods

### 2.1. Cultures

Animal handling was in accordance with the guidelines of the Institutional Animal Care and Use Committee of the Weizmann Institute and with the Israeli National Guidelines on Animal Care. Cultures were prepared as detailed elsewhere [19, 20]. Briefly, E19 rat embryos were removed from pregnant decapitated mother's womb under sterile conditions. The hippocampi were dissected free and placed in a chilled (4°C), oxygenated Leibovitz L15 medium (Gibco) enriched with 30 mM glucose and gentamicin (Sigma, 20 *μ*g/ml) and mechanically dissociated. About 10^5^ cells in 1 ml medium were plated on 13 mm circular glass coverslips in each well of a 24-well plate. Cells were left to grow in the incubator at 37°C, 5% CO_2_.

Neurons were transfected with PS1 or the M146V mutant presenilin 1 (mPS1) and DsRed, GFP, or eBFP2 (to image cell morphology) and mitochondrial or endoplasmic reticulum calcium sensor (red Mt-RCaMP and green GEM-CEPIA1er, respectively), using lipofectamine 2000 (Thermo Fisher Co. 18), at 6-7 days in vitro (DIV), and were imaged at 10-21 DIV. The transfection methodology was adopted from standard protocols [20]. It should be noted that in all experiments parallel imaging was conducted with cells transfected with either PS1 or mPS1, and the fluorescence level was identical in the two groups. Also, transfection yield was similar in both groups (around 1-2% of the cells in a glass).

In some experiments, we imaged transfected PS1 and mPS1 on the background of BFP, followed by fixation of the tissue and immunostaining for PS1. In both groups of neurons, there was a nearly complete overlap of the transfected PS1/mPS1 and the immunostaining for PS1 (Supplementary Figure 1) indicating that the antibody recognizes both transfected varieties of PS1. However, despite extensive experience with transfection methodologies, there is still a paucity of information on either the selective vulnerability of a small proportion of the neurons to transfection as well as the cellular impact of the transfection (see [21]).

PS1 and mPS1 were transferred from the original plasmids to peGFP-C1 and pmcherry-C1 by restriction-free cloning with KAPA HiFi Hotstart ReadyMix (Kapa Biosystems) with the following oligos (Integrated DNA Technologies): 5′-TCTCGAGCTCAAGCTTCGAATTCTGCAGTCACAGAGTTACCTGCACCGTTG-3′ and 5′-CTAGATCCGGTGGATCCCGGGCCCGCGGCTAGATATAAAATTGATGGAATG-3′.

### 2.2. Live Cell Imaging

Cultures were placed in the imaging chamber, on the stage of an upright Zeiss 880 confocal microscope using a 40x water immersion objective (1.0 NA), and imaged at a rate of 10-20 frames/s. No photobleaching was detected under these conditions. Experiments were also conducted on an inverted Zeiss 510 confocal microscope, in specific experiments. Standard recording medium contained (in mM) NaCl 129, KCl 4, MgCl_2_ 1, CaCl_2_ 2, glucose 10, and HEPES 10, pH was adjusted to 7.4 with NaOH and osmolality to 320 mOsm with sucrose. Cultures were incubated with Fluo-2AM (2 *μ*M, Invitrogen) for 1 hour at room temperature to image variations in [Ca^2+^]i resulting from network activity or changes in ambient [Ca^2+^]o. Imaging of cell morphology (401 nm), calcium variations (488 nm), and mitochondrial calcium (550 nm) was made with the appropriate wavelengths. All measurements were conducted with identical laser parameters for all groups (e.g., intensity, optical section, duration of exposure, and spatial resolution).

### 2.3. Mitochondrial Membrane Potential

Cells were cotransfected with eBFP and PS1 or mPS1 (0.4 and 0.7 *μ*g/*μ*l, respectively). Prior to the imaging, cells were incubated with Tetramethylrhodamine Methyl Ester, Perchlorate (TMRM), (100 nM) for 25 minutes in the 37°C incubator. Cells were imaged at 3D (about 1 *μ*m/section, 15-17 sections). Mitochondrial potential was calculated using the formula developed by Koopman et al. [22]:

(1) $$\begin{array}{c} \Delta \psi =-60*\log\left(7.6*\frac{F_{\text{MT}}}{F_{\text{nuc}}}\right). \end{array}$$

### 2.4. Electrophysiology

Recording was made in standard conditions, using patch pipettes containing (in mM) K-gluconate, 136; KCl, 10; NaCl, 5; HEPES, 10; ethylene glycol-bis (beta-amino ethyl ether) N,N,N′,N′-tetra-acetic acid (EGTA), 0.1; Na-GTP, 0.3; Mg-ATP, 1; and phosphocreatine, 5; pH 7.2 with an axis resistance in the range of 5-8 M*Ω*, as described before [23]. Signals were amplified using a MultiClamp 700B amplifier and accumulated and analyzed with PClamp10. Cells were recorded in current clamp mode, and responses to current steps or ramps were recorded and analyzed for passive and active properties. mEPSCs were recorded in the presence of TTX (0.5 *μ*M) and bicuculline (10 *μ*M). In some experiments, a pressure application through a patch pipette of high osmolality medium (addition of 300 mM sucrose to the standard recording medium) was applied near the recorded neuron, to cause and measure an increase in rate/size of spontaneous mEPSC. These data were analyzed using MiniAnalysis and Origin software.

### 2.5. Statistical Analysis

Fluorescence intensity and high-resolution images were analyzed using ImageJ (NIH, USA)- and MATLAB (R2010b, USA)-based programs. Some measurements were made in a double-blind procedure to assure unbiased observations. Statistical comparisons were made with ANOVA followed by *t*-tests, as the case may be, using MATLAB, KaleidaGraph, and Origin software. Statistical significant differences were considered at *p* < 0.05.

## 3. Results

### 3.1. Morphology

**Figure 1:**
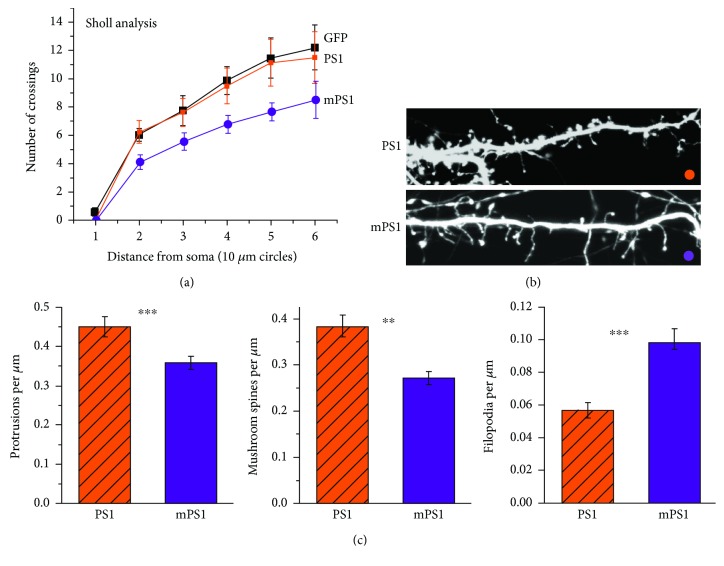
Morphological analysis of dendritic arbors and spines in 14 DIV cultured hippocampal neurons transfected with GFP and PS1 or mPS1. (a) Sholl analysis counting the number of dendrites in 10 *μ*m perimeters around the cell somata. A significant difference was found between mPS1 and the other two groups of neurons. (b) Live images were taken at higher resolution, and the results summarize observations in 30 and 31 dendrites in 10-12 cells in the two groups. Three dimension images were analyzed using ImageJ (see Section 2). The total number of protrusions (c, left) as well as the density of mushroom spines (middle) was significantly lower in the mPS1 group compared to the PS1 group, while the mutant cells expressed higher density of filopodia (long, headless protrusions, right). The differences were highly significant (in this and the following figures ^∗^ *p* < 0.05, ^∗∗^ *p* < 0.01, and ^∗∗∗^ *p* < 0.001).

In the first series of experiments, we analyzed dendritic morphology in 24 GFP transfected neurons (*n* = 8 PS1 neurons, *n* = 9 mPS1, and *n* = 7 control GFP-expressing neurons). Among them were eight neurons cotransfected with PS1, 9 neurons cotransfected with mPS1 species, and 7 control GFP cells. Initially, Sholl analysis was used to estimate the complexity of dendritic branches growing out of the somata of the transfected neurons (Figure 1(a)). While there was no difference between the GFP- and PS1-transfected neurons, there was a significant reduction in the number of branches in the mPS1 neurons, indicating that they are less mature. In subsequent analysis, we compared spine density and shape only in PS1- and mPS1-transfected neurons. The analysis included a total of 30 dendritic segments in the first group and 31 dendrites in the second cohort. The mPS1 dendrites had significantly lower total density of dendritic spines (0.35 ± 0.02 spines per micrometer compared to 0.45 ± 0.02 in the controls), lower density of mushroom spines (0.27 ± 0.01 compared to 0.38 ± 0.02 in the controls) as shown before [24], but a higher density of filopodia (0.1 ± 0.008 compared to 0.05 ± 0.004 in the controls, Figures 1(b) and 1(c), *t*-test, *p* < 0.001), indicating that neurons bearing mPS1 are less mature than their wild-type PS1-expressing counterparts. These results replicate earlier observations [24, 25] on the morphological correlates of mPS1 and justify the continuation of the imaging experiments.

### 3.2. Electrophysiological Analysis

**Figure 2:**
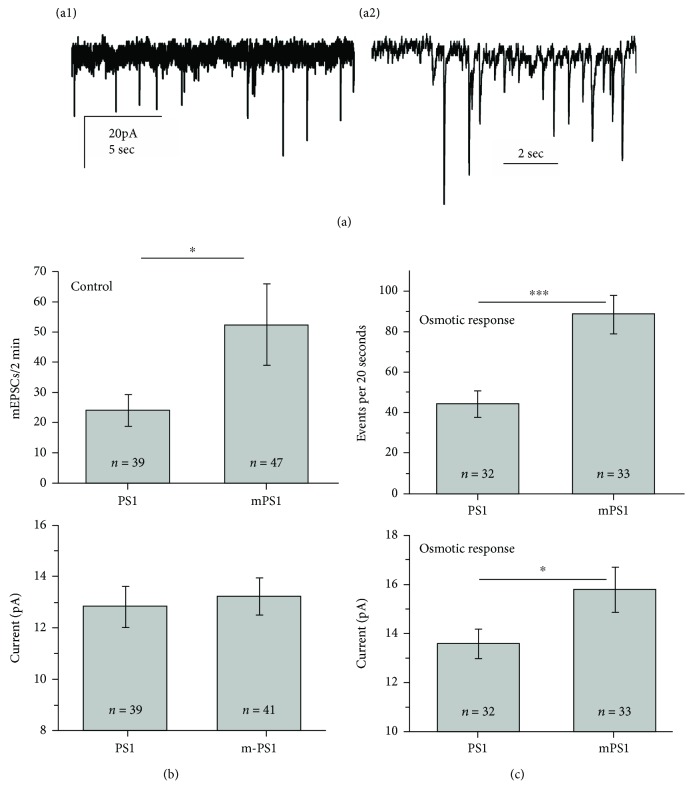
Comparisons of mEPSCs between PS1- and mPS1-transfected neurons. Neurons were recorded in the presence of TTX for 2 minutes, and the number and size of individual mEPSCs were measured and averaged for each cell. Following, in some experiments, the responses to pressure application of sucrose-containing medium were recorded for 20 seconds. (a) A record of mEPSCs in the standard condition (A1) and in response to the sucrose-containing medium (A2). Note difference in time scales between the two records. (b) Comparisons of mean rate (top) and amplitude in the two groups. The rate in the mPS1 group was significantly higher than that in the PS1 group, despite the large variability. (c) In response to hyperosmotic pulses, the values represent mean frequency and size over 20 seconds of recording. A highly significant (*p* < 0.001) increase in mean rates and a significant difference (*p* < 0.05) also in mean size of mEPSCs are obvious.

Comparisons were made between 39 PS1-transfected neurons and 47 mPS1 neurons in 13 different experiments. There were no apparent differences in passive properties of the neurons. Cells were voltage clamped at -60 mV, and mEPSCs were recorded for standard durations of 2 minutes, and mEPSCs analyzed using pCLAMP and MiniAnalysis software. In baseline conditions (Figure 2), there was a small insignificant difference between PS1 and mPS1 cells in the mean magnitude of the events. However, there was a significant difference in the frequency of mEPSC discharges, being higher in the mPS1 cells (*t*-test, *p* < 0.05). In addition, there was no difference in rise time of the mEPSCs between the groups, amounting to 4.1 ± 0.5 ms in the PS1 group and 3.65 ± 0.25 ms in the mPS1 group.

In response to pressure application of high osmolality medium (Figure 2(c), measured in 32 PS1 and 33 mPS1 cells), there was an increase in mEPSC rates and amplitudes in both groups, compared to baseline conditions. This yielded a highly significant difference between the PS1 and mPS1 groups, both in the mean magnitude of events (15.5 ± 0.9 pA compared to 13.6 ± 0.6 pA in the PS1 group, *p* < 0.05) and in the rate of mEPSCs (88 ± 9.3 events per 20 seconds of recording compared to 44.3 ± 6.4 events in the PS1 group, *p* < 0.001, Figure 2(c)). Once again, there was no difference in the time to peak of the events (data not shown). Taken together, these results indicate that the mPS1-containing neurons express normal synaptic connectivity, with significantly higher rates of spontaneous events than controls.

### 3.3. Mitochondrial, ER, and Cytosolic Calcium

Based on the suggestion that PS1 may act at the mitochondria-associated ER membrane (MAM) [2, 24, 26], we explored functional links between PS1, cytosolic calcium concentration, and mitochondria in the following experiments.

Initially, we confirmed that expression levels were similar between PS1- and mPS1-transfected neurons. To this end, we imaged individual neurons in both the somata and dendrites and found similar fluorescence levels, both for GFP expression and PS1 expression in immunohistochemical slides of the same cultures: GFP expression in soma, *n* = 14 cells, fluorescence level (arbitrary units) 181.1 ± 9.4 and 180.9 ± 10.6, in dendrites 51.5 ± 3.8 and 53.1 ± 5.35 for PS1 and mPS1, respectively; immunohistochemical evaluation of PS1 intensity in fixed neurons *n* = 16, soma 23.1 ± 1.3 and 21.8 ± 1.8 in PS1 and mPS1, respectively; in the dendrites of the same cells *n* = 32, 12.2 ± 0.56 and 13.8 ± 0.37 fluorescence units, respectively. There were no significant differences among the groups in any of these parameters. Similarly, when examining PS1-GFP and mPS1-GFP, there was no difference between the groups (data not shown) in the direct expression of the fluorescent plasmids.

**Figure 3:**
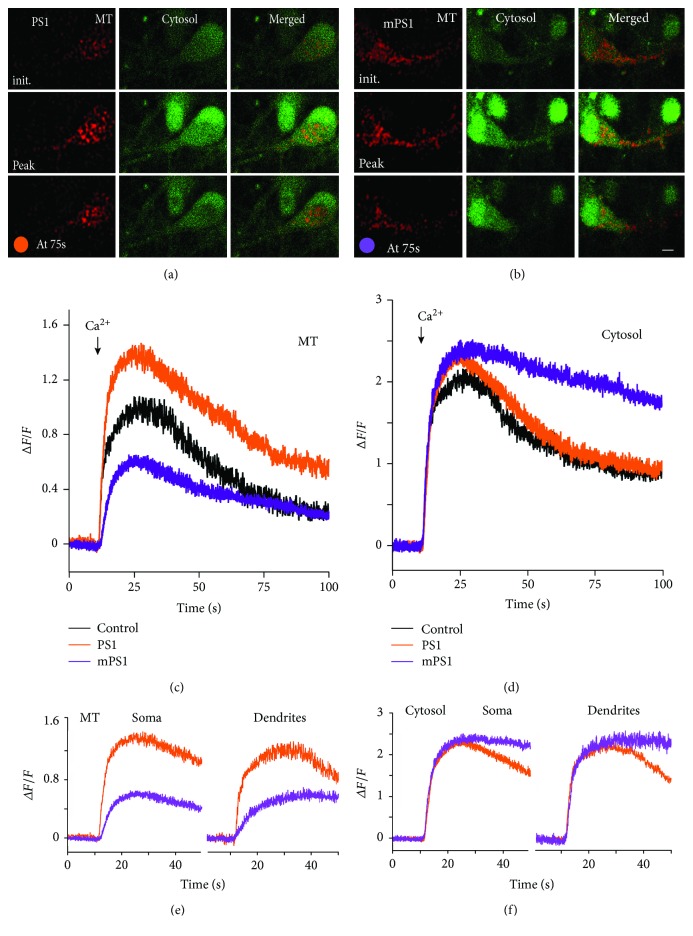
Cells transfected with mPS1 (b) raise mitochondrial calcium ([Ca^2+^]_M_) to lower levels than PS1 (a) in response to a switch in ambient [Ca^2+^]o from 0 (top row, initial) to 2 mM (middle row, peak). Cells were imaged up to 100 s after medium switch (bottom row) to show a recovery from the calcium surge. Cells were cotransfected with eBFP to image morphology, MtRCaMP to image calcium in mitochondria (MT), and PS1 or mPS1. Cultures were loaded with 2 *μ*M Fluo-2AM for 50 minutes prior to imaging, to measure cytosolic calcium. Cells were imaged for 15 minutes after the removal of ambient calcium from the medium and for 100 s after the addition of 2 mM CaCl_2_ to the medium, simultaneously for the mitochondrial and cytosolic calcium at a rate of 20 frames/s. There was a fast increase in [Ca^2+^]_M_ in the somata of the PS1-transfected neurons and a significantly smaller rise in the mPS1-transfected neurons (c). In the same cells, cytosolic calcium raised to the same level in the three groups (d) but recovered much slower in the mPS1 neurons than in the other groups. At least five different locations for each cell were averaged. *N* = control, 10 cells; PS1, 19 cells; and mPS1, 16 cells. (e, f) Same neurons, imaged in the soma and the dendrites, showing similar disparity between the two groups of neurons. For clarity, only the results of the two plasmids are shown, leaving the controls outside the picture. Consult text for details of the analysis.

To examine dynamic changes in calcium concentrations, we initially estimated cytosolic [Ca^2+^]i by Fluo-2 imaging of transfected and adjacent neurons in the nominal absence of calcium in the recording medium, which causes an initial release of calcium from stores, followed by its depletion, to result within 10-15 minutes in a very low level of ambient [Ca^2+^]i. Subsequently, calcium (2 mM) was added to the imaging medium, causing a fast rise of [Ca^2+^]i in all cells, due to a massive activation of calcium uptake through store-operated channels, until the calcium stores are equilibrated and free [Ca^2+^]i returns exponentially to normal ambient levels [17] (Figure 3). The initial rise of cytosolic [Ca^2+^]i was similar in control, PS1, and mPS1 neurons (in the soma, fluorescence level (DF/F) amounted to 1.94 ± 0.19, 2.37 ± 0.20, and 2.31 ± 0.18 in control, PS1, and mPS1, respectively). ANOVA for the three groups yielded no significant difference. Strikingly, the return of [Ca^2+^]i to baseline level was far slower and nonexponential in the mPS1-transfected neurons both in the soma and dendrites (Figures 3(d)–3(f)) compared to the other two groups of neurons.

Simultaneously, we measured mitochondrial [Ca^2+^] ([Ca^2+^]_M_) in the same neurons. [Ca^2+^]_M_ responded slower and reached significantly lower peaks in the mPS1-transfected neurons than the other two groups (Figures 3(c)–3(f)), in the soma (*n* = 15–17 cells in each group) 0.98 ± 0.06, 1.32 ± 0.15, and 0.60 ± 0.05 in control, PS1, and mPS1, respectively. ANOVA followed by Tukey's comparisons yielded highly significant difference (*p* < 0.0001 between the PS1 and mPS1 groups). Similar results were obtained for the dendrites (data not shown). This indicates that the mitochondria of the mPS1 are not as effective in clearing the extra cytosolic calcium elevated by the exposure to calcium ions in the extracellular space. These experiments indicate that the level of cytosolic calcium is higher in the mPS1 cells, probably because calcium is not taken up efficiently into the mitochondria.

**Figure 4 fig4:**
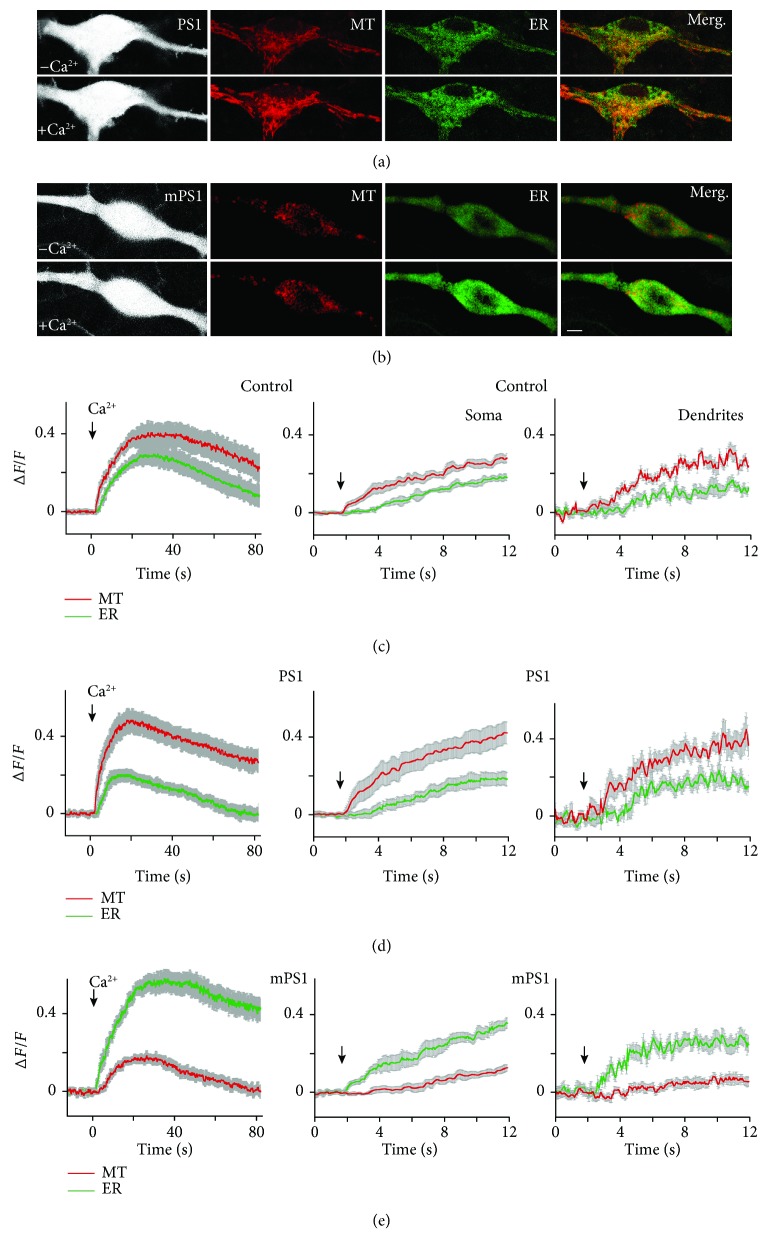
Simultaneous measurements of changes in mitochondrial and ER calcium. (a, b) Cells were cotransfected with ER-GCaMP, MT-RCaMP, PS1 or mPS1, and eBFP at the age of 7-8 days and imaged 2-4 days later. Calcium was removed from imaging medium for 15 minutes and then recovered as above. Imaging was performed using double track mode (no simultaneous illumination), at the rate of 10 frames/s, during about 90 s, using argon (488) and HeNe (543) wavelengths with 40x water immersion objective (NA = 1). At least 5-7 specific locations were averaged for each cell. *n*: 22 control cells, 36 PS1cells, and 32 mPS1cells. (c–e) Left column, measurements in the three groups at the soma region, as indicated. Middle, expanded traces of the onset of the responses to calcium in cells shown on the left. Right column, measurements of the onset of responses in dendrites of the same cells, in an expanded scale.

In a separate series of experiments, we measured changes in [Ca^2+^]_M_ simultaneously with endoplasmic reticulum calcium ([Ca^2+^]_ER_), using two excitation wavelengths linked to the specific plasmids (MT-RCaMP and GEM-CEPIA1er, 550 and 488 nm, respectively). The same stimulation protocol was used as above. While resting calcium levels were similar in the two compartments in all three groups of neurons (Figures 4(a) and 4(b)), [Ca^2+^]_ER_ had faster kinetics and larger peak amplitude than [Ca^2+^]_M_ in the cells harboring mPS1 in response to replenishment of ambient extracellular calcium. The difference was reversed in the PS1-transfected as well as the control neurons, in that the change in mitochondrial calcium was larger and faster than that observed in the ER. A similar disparity was seen in [Ca^2+^]_M_ and [Ca^2+^]_ER_ measured in the dendrites of the imaged neurons, albeit with a larger variability, due to the smaller regions of interest of the imaged dendrites (Figures 4(c)–4(e), right column). At the peak of the response control, [Ca^2+^]_ER_ amounted to (DF/F) 0.29 ± 0.04, PS1 0.172 ± 0.03, and mPS1 0.54 ± 0.04. ANOVA followed by paired comparisons yielded highly significant differences among the group comparisons. In the same groups of neurons at the peak of responses, control MT amounted to 0.385 ± 0.07; PS1-transfected neuron 0.40.06 and mPS1 yielded only 0.177 ± 0.03. Here again, there was a highly significant difference among the groups.

These observations concur with the results of the previous experiment to suggest that mitochondria in the cells harboring mPS1 are less efficient in buffering free intracellular calcium.

**Figure 5 fig5:**
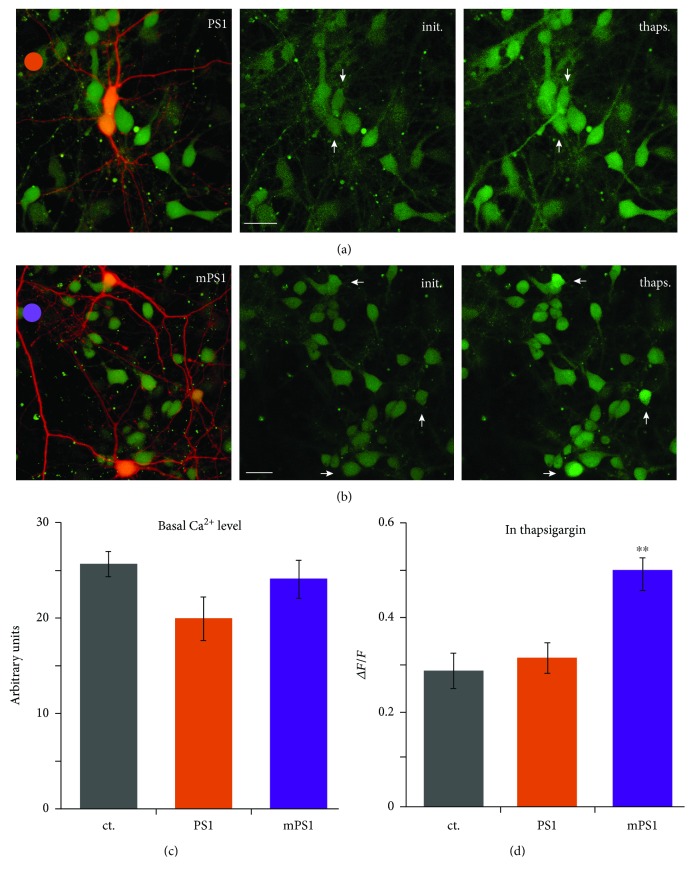
Changes in cytosolic calcium in response to thapsigargin are larger in mPS1-transfected neurons than in the other groups. Cells were transfected with PS1 or mPS1 and DsRed. Cultures were loaded with Fluo-2AM prior to imaging. Single sections were continuously imaged at the rate of 10 frames/s, and thapsigargin (1 *μ*M) was applied to the bath. Maximum responses were used for averaging. (a) PS1-transfected neurons (red) among nontransfected ones. (b) mPS1-transfected neurons. Left images combining the red transfected neurons and the nontransfected Fluo-2-loaded neurons. Middle and right columns in control and in the presence of thapsigargin, respectively. Scale bar 20 *μ*m. Arrowheads point to the transfected neurons. (c, d) Fluorescence levels in baseline where fluorescence is expressed in arbitrary units and (d) during exposure to thapsigargin, where fluorescence is expressed as a change relative to baseline. *n*: control—8 fields, containing 14 transfected cells; PS1—7 fields, containing 14 transfected cells; and mPS1—8 fields, containing 17 transfected cells. mPS1 is highly significant above PS1 and control cells.

To examine the role of the ER in the regulation of [Ca^2+^]i, we exposed the cultures to thapsigargin, a noncompetitive irreversible inhibitor of the endoplasmic reticulum Ca^2+^ ATPase (SERCA) [26, 27]. Neurons were transfected with DsRed and PS1 or mPS1 and were loaded with Fluo-2AM to visualize variations in [Ca^2+^]i. While baseline fluorescence levels were the same in all neurons (Figure 5(c)), following exposure to thapsigargin, the mPS1-transfected neurons raised [Ca^2+^]i to significantly larger amplitude compared to control GFP and PS1 neurons as well as nontransfected neurons visualized in the same field (Figure 5(d)).

Taken together, these experiments indicate that PS1 is instrumental in controlling mitochondrial ability to buffer calcium following a rise of cytosolic calcium, such that in the presence of mPS1, cytosolic calcium elevation is not buffered by uptake into the mitochondria and remains high, unlike the control DsRed and PS1/DsRed-transfected neurons.

### 3.4. Presenilin, Mitochondria, and Oxidative Stress

**Figure 6 fig6:**
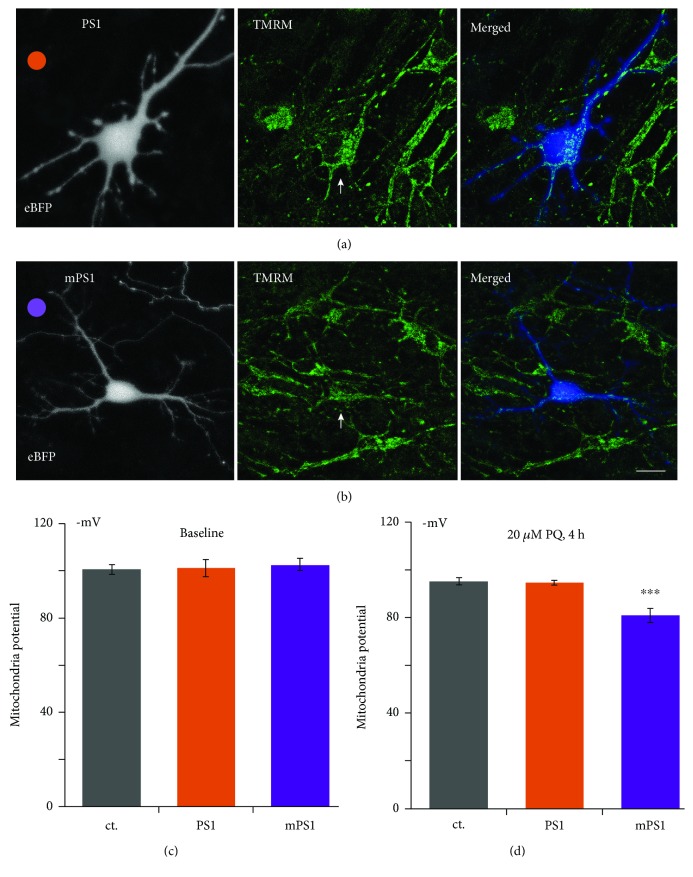
Mitochondrial membrane potential is more depolarized in stressed mPS1-transfected neurons. Cells were cotransfected with eBFP to image morphology and PS1 (a) or mPS1 (b). Prior to imaging, cells were incubated with TMRM (10 *μ*l/ml) for 25 minutes in the 37°C incubator. Cells were 3D imaged (about 1 *μ*m/section, 15-17 optical sections per cell). Paraquat (20 *μ*M) was added to the bath for 4 h. At least 5 MT-containing locations were averaged for each cell. *n*: 6 fields containing 10 transfected cells for each condition in baseline and 8 fields containing 10 control cells, 16 PS1 cells, and 15 mPS1 cells. Images (from left to right): eBFP-transfected neurons to outline the morphology of the cell, TMRM fluorescence to image mitochondrial potential, and a merged image (right). Scale bar 5 *μ*m. (c) In resting conditions, no difference among the three groups of neurons. (d) During exposure to paraquat (PQ) for 4 hours, showing more depolarized potentials in all three groups compared to baseline, but the largest effect was seen in the mPS1 group (*p* < 0.00012).

To examine the functional consequences of the dysregulation of [Ca^2+^]_M_ in the mPS1, we measured mitochondrial membrane potential, using TMRM, in both baseline conditions and following exposure of the cultures to paraquat (PQ), an oxidative stressor that impairs mitochondrial membrane permeability (20 *μ*M, added for 4 hours prior to imaging, Figure 6). There was no apparent difference in mitochondrial potential among the three groups of transfected neurons at rest (amounting to −100.8 ± 2.09 mV, −101.4 ± 3.37 mV, and −102.6 ± 2.71 mV in control, PS1, and mSP1, respectively). Strikingly, the drug caused a significant elevation of mitochondrial potentials in all neurons to −95.1 ± 1.68 mV and −94.7 ± 0.84 mV in the control and the PS1-transfected neurons, but the mPS1-transfected neurons were affected significantly more than the other two groups (to −80.9 ± 2.91 mV) (ANOVA, *F* = 12.041, df 35/2, *p* < 0.00012 (Figure 6(d))).

**Figure 7 fig7:**
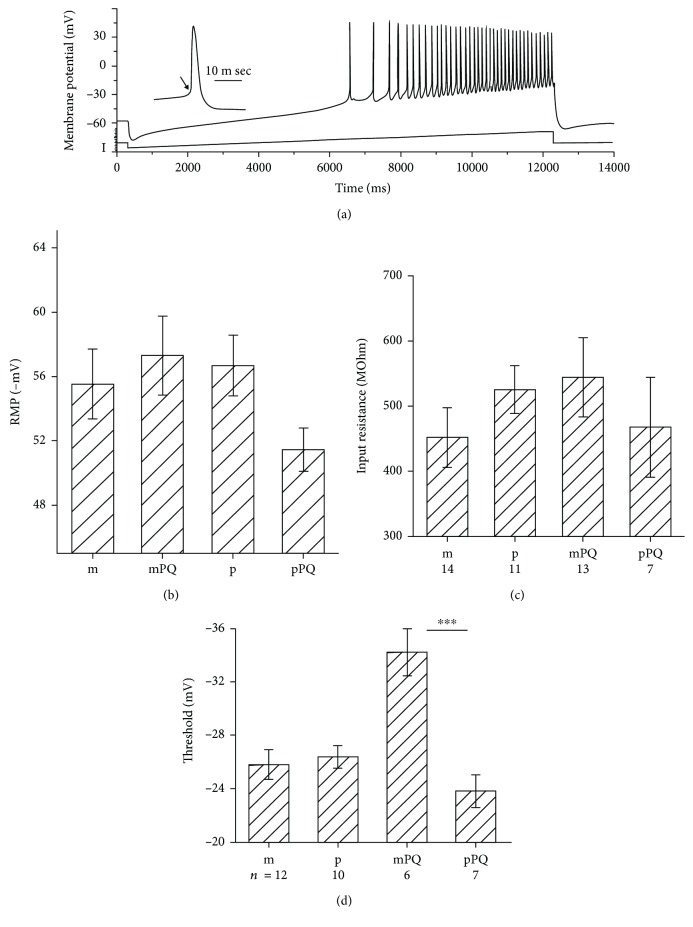
Paraquat (PQ) causes an increase in excitability of mPS1-transfected neurons. (a) Sample illustration of a current clamped neuron, responding to a slow current ramp lasting 12 seconds, from below resting potential to a depolarizing potential. The cell begins to discharge action potentials around -30 mV and continues to fire at gradually higher rate until return to resting potential. Spike threshold can be measured (arrowhead) as well as spike size and after potential. (b–d) Averages of resting membrane potential (b) input resistance (c) and spike thresholds (d), in mPS1 (m), mPS1 in the presence of PQ (mPQ), PS1 (p), and PS1 in the presence of PQ (pPQ). A large and highly significant change in spike threshold is obvious.

Possible functional consequences of a 1-1.5 h exposure to paraquat were examined in patch-clamped neurons recorded in current-clamp mode, to assess difference in spontaneous activity as well as in response to depolarizing current pulses (Figure 7). Responses to short current pulses were measured, as well as responses to a current ramp, from below resting potential to a marked depolarization which produced a continuous barrage of action potentials (Figure 7(a)). Recording from cells exposed to PQ was done in the presence of the drug in the recording medium. Four independent groups of neurons were used. These included mPS1, PS1, mPS1/PQ, and PS1/PQ, in 6-10 experiments. There was no difference in resting membrane potential (Figure 7(b)), input resistance (Figure 7(c)), and after hyperpolarizing potentials (AHP, data not shown) among the groups. In contrast, there was a highly significant decrease in spike threshold in the mPS1/PQ-treated cells (ANOVA, df = 3/64, *F* = 7.06, *p* < 0.001, followed by *t*-test, *p* < 0.001 comparing mPS1PQ with PS1PQ groups) meaning that the mPS1 cells discharged action potentials at lower thresholds, making them more excitable than those of the other groups.

**Figure 8:**
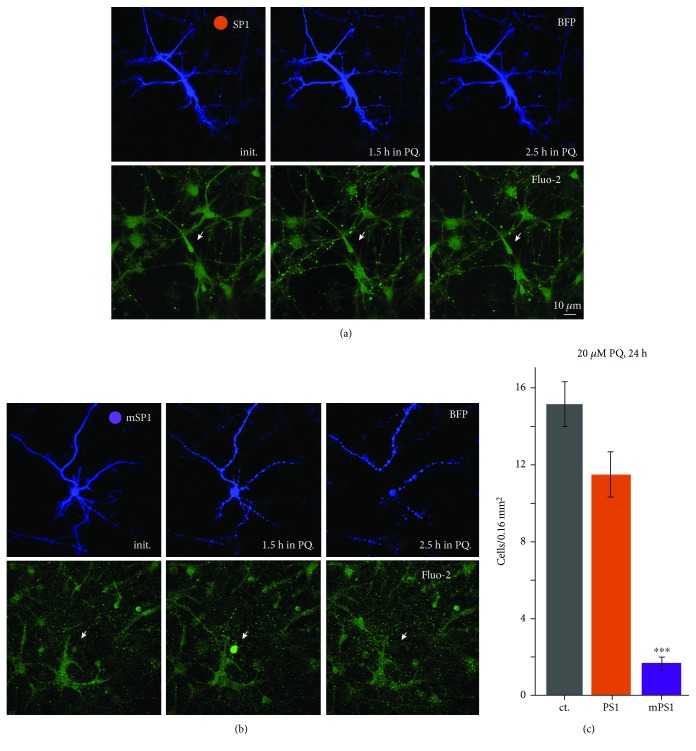
mPS1-transfected neurons are more sensitive to oxidative stress than controls. Cells were transfected with eBFP and PS1 or mPS1 and treated with paraquat (20 *μ*M) for 24 h in the incubator. For each group, the entire glass was imaged on confocal microscope using tail mode (see Section 2 for details). All transfected cells in the glass were counted using 400 × 400 *μ*m squares and the number obtained was averaged for each glass. *n*: 8 glasses for each group. A marked reduction in the mPS1-transfected neurons per glass was evident (*p* < 0.0001).

The inability to maintain mitochondrial membrane potential in the presence of an oxidative stressor may affect reactivity to afferent stimulation and eventual viability of the neurons [28]. We used a modest concentration of the mitochondrial stressor, paraquat, to compare mPS1- with PS1-transfected neurons. Cultures were exposed to 20 *μ*M paraquat for *24* hours, and the fluorescently tagged neurons in the different culture dishes were counted (Figure 8). In this experiment, the entire glasses were imaged on the confocal microscope using “tail mode,” and all transfected neurons in a glass were counted in 400 × 400 *μ*m squares. The number of transfected cells for each glass was averaged in 8 glasses per condition. There was a highly significant reduction in paraquat-treated, mPS1-transfected neurons, compared to the other two paraquat-exposed groups (*n* = 8 glasses for each group; 15.2 ± 1.17, 11.5 ± 1.18, and 1.7 ± 0.33, ANOVA; *F* = 88.79, df 23/2, *p* < 0.0001) as well as the control, drug-free conditions (not shown).

These experiments indicate that while the mitochondria of mPS1-transfected neurons are less efficient in buffering a rising ambient [Ca2+]i, the cells are unable to withstand oxidative stress, leading to their eventual degeneration.

## 4. Discussion

Taken together, our results confirm and extend earlier observations on functions of PS1 in cultured hippocampal neurons. Briefly, mPS1-transfected neurons possess lower density of mature, mushroom spines compared to wild-type PS1-transfected neurons. However, this difference is associated with a higher frequency and amplitude of mEPSCs following exposure to hyperosmotic recording medium. The discrepancy between the morphological results suggesting an immature neuron (Figure 1) and the electrophysiological ones indicate that even though the cells are immature, they do receive synaptic inputs from adjacent normal nontransfected cells. However, these synapses are likely to be located on dendritic shafts, as suggested before [23]. Neurons transfected with mPS1 raise [Ca^2+^]_M_ to lower level than controls, following replenishment of [Ca^2+^]o. These results indicate that mPS1-containing neurons are less efficient in reducing [Ca^2+^]i as indicated by its sustained elevation, which is detrimental to cell viability, and consequently, these neurons have higher excitability and lower viability when exposed to oxidative stress.

Interestingly, both presymptomatic AD mice model and human patients express hyperexcitable central neurons. In the mouse model, hyperexcitability is assumed to result from the downregulation of the potassium channel KV4.2, which underlies a transient voltage-gated potassium current [29]. In humans, susceptibility to epileptic seizure is one of the early predictors of AD, and this reflects an increase in excitability [30].

Our results confirm earlier observations [31] suggesting that mPS1-containing neurons express fewer mushroom spines and higher density of filopodia, the immature protrusions. This indicates that the affected neurons are less able to form stable synapses. However, we found that these neurons express higher rates of miniature synaptic currents, unlike previous observations made in homogeneous population of neurons in a microisland [32]. The discrepancy between these two sets of observations may reflect the fact that in our case only a minority of neurons are transfected in otherwise normal dissociated cultures. Since the transfected neurons are innervated mostly by untransfected ones, the synaptic connections may be different from the case where all neurons are endowed with the mutant PS1. For example, presynaptic terminals, which contain mitochondria, are also likely to be affected by the mPS1 to produce a reduction in release probability. This has to be explored in further experiments. Obviously, this was not a case with our cells, where in response to high osmolality, transfected mPS1 neurons increased rates of mEPSCs more than wt PS1 neurons. Indeed, the enhanced reactivity to hyperosmotic medium is assumed to reflect an increase in release probability, but this can also result from activation of otherwise nonactivated postsynaptic receptors. The larger [Ca^2+^]i response to caffeine, seen elsewhere [14, 15, 33], indicates that mPS1-endoplasmic reticulum stores contain more releasable calcium pools than control cells, indicating a possible presynaptic locus of action.

These results may explain the schism between the first (Figures 1 and 2) and the second (Figures 3 [Fig fig4] [Fig fig5] [Fig fig6] [Fig fig7]–8) sets of results observed herein; if indeed there is a break in the MAM connecting the ER to mitochondria, it is likely that calcium is accumulating in the ER, not being able to move to the mitochondria. Under these conditions, ER-dependent functions will be enhanced, causing higher [Ca^2+^]i transients in response to caffeine and higher rates of mEPSCs, while mitochondria-dependent functions may be suppressed. This hypothesis should be explored directly in further experiments.

One other discrepancy between our results and previous ones, suggesting that there are more mitochondrial-ER connections in the mutant PS1 [1], may be related to the fact that our cells are transfected with the mutant PS1 in the presence of the native species, which was not suppressed prior to the transfection. Since the antibody that we have used cannot distinguish between native and transfected PS1 and the detected level of PS1 is the same as in the nontransfected neurons, we assume that the native one is downregulated in the transfected neurons. This assertion has to be verified in future experiments.

mPS1 has been shown to compromise the process of neuronal store-operated calcium entry (nSOC) in cultured hippocampal neurons from PS1M146V knock-in mice due to downregulated STIM2, an ER calcium sensor and an activator of Orai channels, while activation of nSOC pathway rescued mushroom spine loss in these mice [27]. This observation may be secondary to the association of PS1 with mitochondria [29, 34], in that depletion of calcium from ER may recruit the STIM/Orai pathway. Evidence for the primary role of PS1 in calcium entry through the Orai channel exists in parallel with evidence for the role of PS1 in MAM regulation of mitochondrial calcium and the consequent depleted calcium functions in the mitochondria [34]. As mentioned above, it is not clear which of the two functions is primarily impaired in the mPS1 neurons. In the long run, the lack of dynamic regulation of [Ca^2+^]_M_ will lead to synaptic disconnection and ultimate neuronal death.

Likewise, since both mutations in PS1 and PS2 are linked to fAD, it is not clear what the critical role of the two presenilins is, and it has been suggested that PS2 is the one that regulates MAM [35]. Further experiments are needed to comprehend the exact nature of the role of PS1 in mitochondrial functions and if indeed mutation in the gene for PS1 leads to neuronal death and consequently Alzheimer's disease.

## 5. Conclusions

In this study, we describe the effects of a mutated PS1 on calcium signaling and synaptic and mitochondrial functions in cultured hippocampal neurons. Neurons harboring the mutated PS1 express more synaptic currents and higher excitability but lower ability of mitochondria to survive a surge of oxidative stress.

## Data Availability

All the generated data and the analysis developed in this study are included within this article.

## Supplementary Materials

Supplementary Materials Supplementary Figure 1: (A) cells transfected with GFP alone, left; GFP+PS1, middle; and GFP+mPS1, right; fixed and immunostained for PS1 (red). (B) Analysis of immunostaining intensity (artificial units) in the cytosol (excluding nuclei) of the transfected neurons. *N* = 17 neurons in each group, staining intensity for GFP alone 23.9 ± 1.57, for PS1 33.0 ± 1.19, and for mPS1 25.1 ± 1.39. ANOVA *F* = 12.6, *p* < 0.0001, Tukey's comparisons show significant difference between PS1 and the other two groups. (C, D) Immunostained PS1 recognizes the fluorescent PS1/mPS1 plasmids in the same dendrites. Top, transfected neurons with BFP and PS1 (left) or mPS1 (right), both linked to GFP. The transfected PS1 alone is seen in the bottom frames in green. In the middle, immunostaining for PS1 (red) and the merged image of the immunostained and transfected species show nearly complete overlap.
