# Supplementary figures and images for: Presenilin 1 Regulates [Ca2+]i and Mitochondria/ER Interaction in Cultured Rat Hippocampal Neurons
Source: Oxid Med Cell Longev. 2019 Jul 28;2019:7284967. doi: 10.1155/2019/7284967 (PMC6701405; doi:10.1155/2019/7284967)

Supplementary Figure 1:

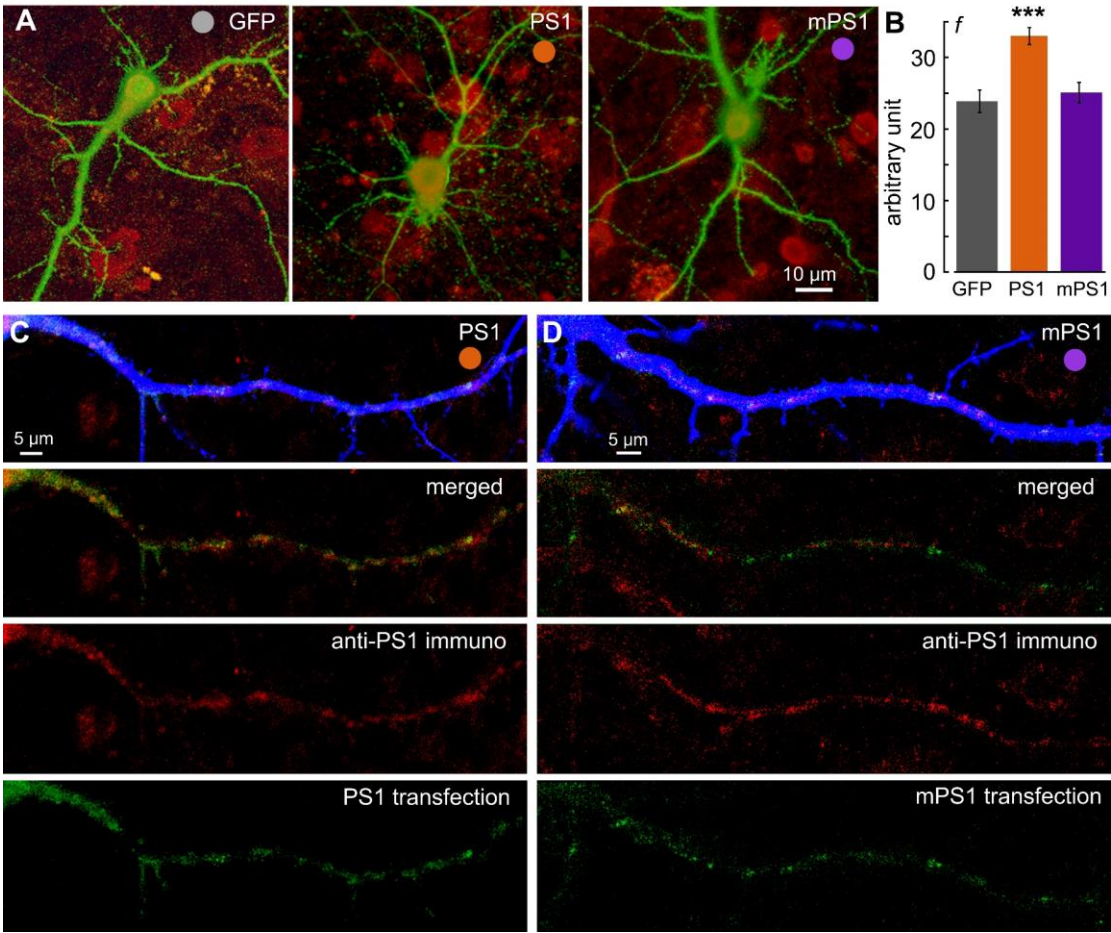

Supplement: Supplementary Materials — Supplementary Figure 1: (A) cells transfected with GFP alone, left; GFP+PS1, middle; and GFP+mPS1, right; fixed and immunostained for PS1 (red). (B) Analysis of immunostaining intensity (artificial units) in the cytosol (excluding nuclei) of the transfected neurons. N = 17 neurons in each group, staining intensity for GFP alone 23.9 ± 1.57, for PS1 33.0 ± 1.19, and for mPS1 25.1 ± 1.39. ANOVA F = 12.6, p < 0.0001, Tukey's comparisons show significant difference between PS1 and the other two groups. (C, D) Immunostained PS1 recognizes the fluorescent PS1/mPS1 plasmids in the same dendrites. Top, transfected neurons with BFP and PS1 (left) or mPS1 (right), both linked to GFP. The transfected PS1 alone is seen in the bottom frames in green. In the middle, immunostaining for PS1 (red) and the merged image of the immunostained and transfected species show nearly complete overlap. [file 7284967.f1.pdf]
